# Targeted antimicrobial use admission provides an actionable denominator for evaluating inpatient length of therapy

**DOI:** 10.1017/ash.2025.10119

**Published:** 2025-09-29

**Authors:** April Dyer, Elizabeth Dodds-Ashley, Angelina Davis, Melissa Johnson, Travis Jones, Rebekah W. Moehring

**Affiliations:** 1 Duke Center for Antimicrobial Stewardship and Infection Prevention, Durham, NC, USA; 2 Duke Antimicrobial Stewardship Outreach Network (DASON), Durham, NC, USA; 3 University of North Carolina Medical Center, Chapel Hill, NC, USA

## Abstract

Our novel antibiotic use denominator, targeted antimicrobial use admission, is defined as an inpatient admission in which a select agent or group of agents is administered. Used in combination with length of therapy, it allows programs to quickly assess agent inpatient durations.

Excess antibiotic days may be attributed to inappropriate empiric therapy or excessive treatment durations.^1^ Accessible data and analysis methods are needed to both develop an optimal stewardship strategy and then measure the impact of that work.

Most stewardship programs use days of therapy (DOT) per 1,000 days present to measure antibiotic use. DOT is the number of calendar days a patient receives an antibiotic, with each agent counted individually. The denominator, 1,000 days present, allows stewards to compare use among hospitals or units with different patient volumes.^2^ Although helpful for benchmarking to recognize higher than expected rates, this metric does not delineate between excessive new starts versus durations of therapy, which require different stewardship strategies. For example, longer durations could be addressed with prospective audit and feedback, while empiric choice or new starts could be affected by the ordering process or restriction criteria. To more quickly target and develop our stewardship strategies within the Duke Antimicrobial Stewardship Outreach Network (DASON),^3^ we developed a metric we call targeted antimicrobial use admission (TAUA).

## What is TAUA?

**Figure 1.**
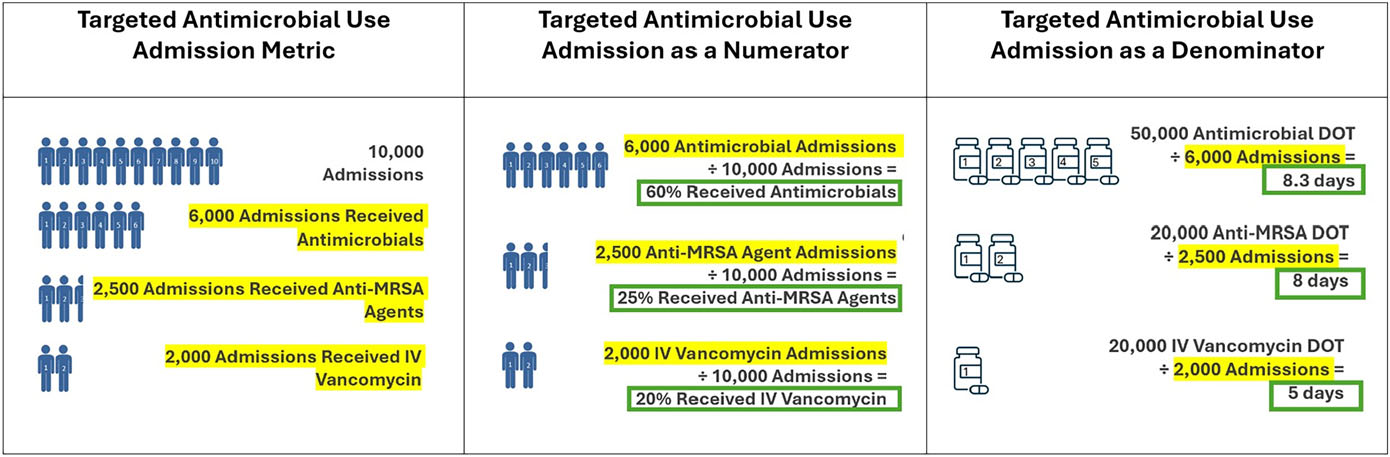
Targeted antimicrobial use admission calculated among all antimicrobial agents, anti-MRSA agents, and IV vancomycin. Each blue figure represents 1,000 admissions. Each blue pill bottle represents 10,000 DOT.

TAUA is simply the count of admissions that receive an antimicrobial agent (or agents) of interest. The antimicrobials of interest may be all antibacterial agents, specific agent groups (eg, anti-MRSA agents), or individual agents. The metric is useful as both a numerator, to determine the percent of patient admissions receiving targeted antimicrobials, and a denominator, where average durations can be calculated if total days (individual agent) or length of therapy (LOT) (agent groups) are known (Figure 1). To better understand how these metrics are distributed among a comparative network, let’s assess IV vancomycin use among 25 hospitals in DASON for calendar year 2017. The mean and standard deviation, median, and range were calculated for different metrics: DOT, (5,147 ± 2,994; 5,093; 512–13,026); DOT/1,000 patient days, (111 ± 24.5; 103.5; 68.9–163.6); DOT/TAUA, (3.2 ± .5; 3.2;2.6–4.2). DOT in aggregate does not allow delineation to the patient level. As previously discussed, DOT/1,000 patient days provides an indication of how “high” or “low” IV vancomycin use is compared to other facilities but does not help the steward select an actionable intervention. However, DOT/TAUA allows the stewardship program to quickly assess the durations of IV vancomycin for admissions receiving this drug. In the example, the mean duration is 3.2 days, which can be used to compare individual patients to this average during chart reviews or compare their average duration to the network mean. If you prefer to evaluate durations for an agent group, you will substitute LOT/TAUA for DOT/TAUA.

Next, let’s review case examples from DASON where TAUA was used to drive antimicrobial use improvements.

## TAUA as a denominator

Hospital networks can use benchmarking data containing the LOT/TAUA metric to determine if durations of therapy are a good target for specific hospitals and to develop an implementation strategy that has greater impact. The 2015 benchmark found a DASON network mean LOT/TAUA of anti-MRSA agents of 3.2 days. Hospital Y had an average duration of 4.1 days and decided to implement a timeout intervention on day 3 (as opposed to day 5) to ensure most patients receiving the antibiotic would be affected by the timeout.

## TAUA as a numerator

Hospitals can benchmark the percentage of inpatients who receive antibacterial agents to evaluate if empiric prescribing is a driving force of above-benchmark antimicrobial use. In 2018, 61.22% of inpatients at Hospital T were receiving an antibacterial agent compared to 57.34% of inpatients among DASON hospitals. Hospital T set a goal to reduce empiric antimicrobial use, and educated providers on instances where antimicrobials could be avoided (eg, asymptomatic bacteriuria, upper respiratory infections), improved diagnostic stewardship (eg, MRSA PCR nasal swabs, updating urine reflex criteria), and worked with infection prevention to reduce culture contamination. These interventions led to a steady decrease in percent of inpatients with antibacterial use, ultimately achieving a percent (51.73%) below the 2022 DASON benchmark (54.94%).

## Can reducing empiric use of agents increase durations?

In 2018, Hospital W noted the percent of patients receiving ertapenem was higher than DASON mean (3.71% vs 1.15%), but durations were lower (3 vs 3.3 d). Hospital W began pharmacist reviews of orders for ertapenem for appropriateness and duration prior to dispensing. Surgeons and hospitalists were educated to avoid ertapenem use and received biannual feedback reports comparing their ertapenem use to peers. These interventions led to a notable decrease in empiric ertapenem use from 2018 to 2019 (3.71% to 0.94% of admissions receiving ertapenem), but durations of therapy increased (3 to 3.44 d). We attributed these increased durations to reducing empiric short-duration surgical use and more appropriate selection of patients who required ertapenem therapy for pathogen-targeted therapy. This example highlights the importance of evaluating these metrics relative to overall antimicrobial use trends.

## Strengths and limitations of TAUA

TAUA is easily captured from the electronic administration record and available to many stewardship programs, although not in the NHSN aggregate data. Combining TAUA with readily available data such as DOT and total admissions helps identify the reason behind “high” antibiotic use rates and aids the stewardship team in developing actionable interventions with a higher likelihood of success. These data are useful for evaluating hospital-specific use and benchmarking for all antibacterial agents, specific agent groups), and individual agents.

The TAUA metric data presented here required patient admission level data sets shared among a hospital network, which may not be accessible for all programs. Further, DOTs are limited to inpatient prescribing and do not capture total durations (inpatient plus postdischarge durations). Thus, hospital length of stays (LOS) can affect duration estimates when TAUA is used as a denominator and postdischarge DOT are not counted. To address this limitation, we recommend using DOT/TAUA (average duration) metrics within the context of overall antimicrobial use rates and a baseline knowledge of how average hospital LOS compares to other network hospitals. In our routine assessments for DASON, we first evaluate overall rates in DOT/1,000 days present and then look to the TAUA/total admissions percent and DOT/TAUA (average duration) metrics as a next step to further describe the pattern of use. If a hospital has average or low DOT/1,000 days present but longer duration estimates, in some cases they also have longer average hospital LOS and a case mix of special populations (eg, oncology).

Prescriber-specific metrics utilizing TAUA may be a future opportunity; however, prescriber-level admission and DOT is not as easily captured from the electronic record due to the complexity of provider attribution by calendar day and practice models for team-based care that include handoffs and shared responsibilities. Future research could focus on capturing data required to expand TAUA to prescriber- and indication-based estimates. Efforts to combine data sources to count both inpatient (electronic medication administration records) and postdischarge (electronic discharge prescriptions) antibiotic days to measure total durations attributed to the hospital stay are already underway.^4–6^

We describe our use of TAUA to create two simple metrics that help us develop stewardship strategies, which are particularly useful when provided within the context of a network benchmark. We believe this combination of metrics help identify the greatest intervention opportunities. Accessible metrics of antibiotic use on the admission level, interpreted within context, can provide actionable data for stewardship programs to design and track interventions.
